# Supplementation and Mitigating Cognitive Decline in Older Adults With or Without Mild Cognitive Impairment or Dementia: A Systematic Review

**DOI:** 10.3390/nu16203567

**Published:** 2024-10-21

**Authors:** Qi Fu, Jill DeJager, Elizabeth M. Gardner

**Affiliations:** Department of Food Science and Human Nutrition, Michigan State University, East Lansing, MI 48823, USA; fuqi@msu.edu (Q.F.); dejagerj@msu.edu (J.D.)

**Keywords:** dementia, cognitive decline, Alzheimer’s disease, aging, nutrition, supplement, vitamins, PUFA, probiotics

## Abstract

This systematic literature review aims to answer the question of how micronutrients might influence the development and progression of dementia. In the present work, we focused on an overview of an updated review of relevant literature published in the last two decades. This review aims to delineate the relationship between micronutrient supplementation and cognitive decline in older subjects. In carrying out this review, we followed PRISMA, and our literature search was performed on PubMed. This systematic review includes only primary studies that have investigated the efficacy of nutritional interventions for the prevention of dementia and improvement of cognitive function in subjects aged 65 years or older with normal cognition, mild cognitive impairment (MCI), or Alzheimer’s disease (AD). A gross heterogeneity of studies forbids the possibility of a direct comparison of the results. A review of the inclusion criteria and restrictions has been conducted to check the validity and reliability of the results. In this review, thirty-three primary studies were included. Results have shown that supplementation with vitamin D, probiotics, and PUFAs would most likely reduce cognitive decline, dementia, or AD compared with vitamins A, B, C, and E, which were seen to be relatively ineffective. Of note, when considering vitamin B supplementation, positive effects were only observed in non-aspirin users having high ω-3 fatty acid (ω-3 FA) plasma levels. In some cases, however, there were genotypic differences in subjects in response to vitamin B supplementation.

## 1. Introduction

With the population aging, chronic conditions have become a burden to healthcare systems [1]. Dementia is perceived to be a severe public health problem, as there are no available effective treatments in the market as of now. In January 2024, nearly 7 million people aged 65 and older were diagnosed with AD, the most common type of dementia, which remains the fifth leading cause of death in the United States [2,3]. Further, the World Health Organization (WHO) has projected that because of global societies’ aging and increased longevity, the number of older adults living with dementia will grow from 46 million in 2015 to 152 million in 2050 [4]. In fact, according to scientists, AD and many other dementias can be avoided, or at least postponed [5], as it is believed that about 40% of the incidence could be avoided [6].

There is accumulated evidence suggesting that different nutritional components may have roles in reducing the significant risk of dementia. Regarding the role of reactive oxygen species (ROSs) in neuronal damage associated with AD, research for this decade indicates that ROSs may play an essential role in it. Vitamins perform many physiological functions and protect the brain through multiple mechanisms, which include protection from the harm caused by free radicals. For example, research has focused on vitamin A’s role in controlling amyloid plaque formation, one of the hallmarks of AD [7,8]. Vitamins C and E are associated with dementias because they act as antioxidants that maintain neurotransmitters [9,10]. B vitamins have some critical roles in the brain: neurotransmitter synthesis, maintenance of nerve cells, and adjustment of homocysteine levels [11,12]. Vit-amin D is reported to have functions that modulate the integrity of the blood–brain barrier and decrease neuroinflammation [13,14,15]. Given the large variety of functions described above, it is reasonable to suggest that vitamins could influence the pathophysiological processes of dementia development in the central nervous system (CNS). In addition, polyunsaturated fatty acids (PUFAs), especially ω-3 FAs, like eicosapentaenoic acid (EPA) and docosahexaenoic acid (DHA), are among other nutritional factors considered as crucial for maintaining neuronal structure and function [16,17]. Probiotics have also been related to dementia through their effects on gut health and probable influence on brain function via the gut–brain axis [18,19,20].

Although many scientific studies have explored the potential of supplements in the preservation and promotion of cognitive health, it remains uncertain whether supplementation influences delaying cognitive decline exclusively in older subjects. Many observational studies and randomized controlled trials (RCTs) reviewed included both middle-aged and older adults. However, by definition, those aged 65 years and older are generally considered as older adults, while those aged from 45 to 64 years are considered as middle aged [21]. Thus, this literature review only examined data on cognitive performance for individuals 65 years and older and included studies conducted over the past two decades. The comparisons to the study limitations and bias were assessed and synthesized to summarize the current evidence in aging brains and the growing evidence that links specific micronutrients with cognitive dysfunction. The present review focused on identifying the underlying cognitive effects of supplementation and intervention, including the consumption of micronutrient supplements, such as vitamins A, C, E, B, and D; probiotics; and PUFAs, like EPA and DHA. According to these pooled data, the associations of micronutrients with prevalent dementia and cognitive impairments in older individuals were assessed.

## 2. Materials and Methods

### 2.1. Search Strategy

The role of supplementation in the development of cognitive decline and dementia has been the subject of several recent systematic reviews that formed the basis for the cur-rent review. The PubMed database was searched from 2004 to 2024 to identify studies published within the past 20 years. The search strategies used text words and relevant indexing to capture studies investigating the association between nutrition and cognitive impairment. Key search terms included text-based terms: “MCI” OR “dementia” AND “older” AND “antioxidant supplement” OR “PUFA” OR “vitamin” NOT “review.” The study selection process consisted of primary screening for eligibility and secondary screening for specific cognitive outcomes. To be included in the primary screening, studies needed to meet all the following criteria: (a) published within the last 20 years (between 2004 and 2024); (b) human beings only; (c) older adults of any gender aged at least 65 years; (d) primary studies only; (e) published in English; (f) accessible published full articles. The purpose for excluding studies before 2004 was to keep up to date with current research findings. There was no restriction on weight or comorbidities, given that subjects often function as a comparison group in controlled trials. Only studies published in English were included because of limited translation resources and limited access to foreign websites. To understand the effects of specific supplements for the secondary screening, the dependent variables must belong to one of the following categories: vitamin A, vitamin C, vitamin E, vitamin B, vitamin D, PUFA, or probiotics. It is worth noting that because of limited information pertinent to vitamins A, C, and E, we broadened the search criteria to include studies involving individuals aged 55 and older. Additionally, a secondary analysis was included when we analyzed PUFAs related to supplementation’s insightful and valuable contributions.

### 2.2. Study Selection Process

**Figure 1:**
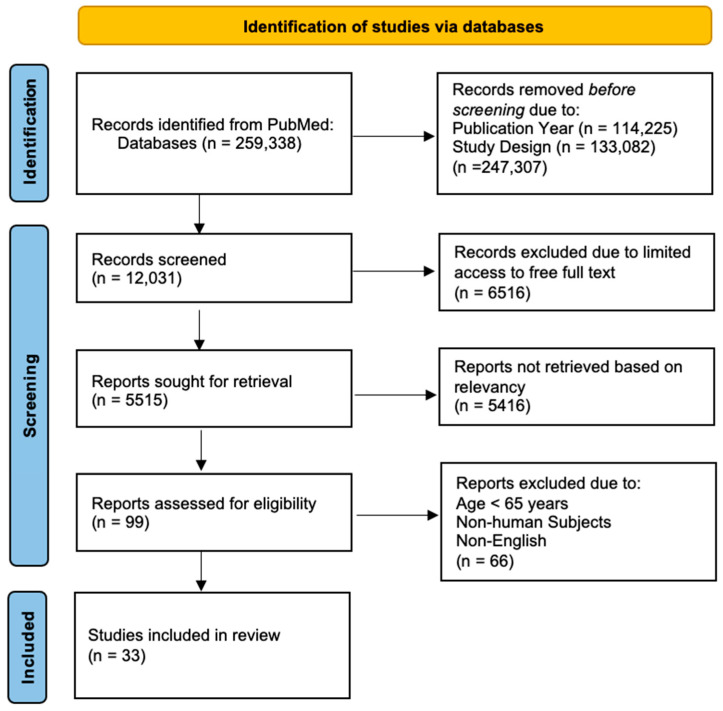
Eligibility criteria for the study of supplementation and mitigating cognitive decline in older adults with or without mild cognitive impairment or dementia: a systematic review. This graph is guided by the PRISMA 2020 flow diagram.

The PRISMA specifications guided this systematic review to compile relevant evidence. The literature review flow diagram shown in Figure 1 is based on the PRISMA 2020 Flow Chart adapted for the scoping review process. This search strategy resulted in 259,338 studies through the PubMed database. Of these, 5515 were selected based on the free full texts, and 99 were selected based on the text relevancy. In total, 33 studies fulfilled the inclusion criteria for the final analysis (Figure 1). For each included study, the following data were extracted: (1) first author and publication year; (2) main result (relationships shown in arrows); (3) dependent variable if the trial was randomized and controlled or study method if the study was observational; (4) sample size of participants; (5) baseline mental status (non-demented, MCI, or AD); (6) age range; (7) measurements for cognitive outcomes; (8) length of the study.

## 3. Results

### 3.1. Vitamins

#### 3.1.1. Vitamins A, C, and E (Antioxidants)

It has been postulated that AD pathogenesis is mediated by oxidative stress [22,23]. Antioxidants represent a group of substances that inactivate free radicals by transforming them into less-reactive compounds and include fat-soluble vitamins A and E and the water-soluble vitamin C [24]. From this theory, this may modulate the adverse effects of oxidative stress on cognitive performance and present a potential opportunity to slow the progression of dementia. Our literature review did not find any study that met our inclusion criteria. However, it would be valuable to examine the existing research on antioxidant consumption, whether from serum contents or supplements, to examine existing findings. In a randomized, double-blind, placebo-controlled trial, there were no effects of vitamin E supplementation on the prevention of dementia, especially in men over 60 years old, after a follow-up period of 6 years [25]. This conclusion is further underpinned by three other studies that drew upon data regarding the use of self-reported supplements and serum levels of vitamin E [26,27,28]. One of these lasted for 15 years and measured the self-reported use of vitamins C and E and found no relationship between supplementation and delay in the incidence of dementia or AD [28]. Similarly, Koch et al. described that the levels of plasma antioxidants were not significantly connected to incident dementia or AD in persons over 65 [26]. However, it partially contravenes the conclusions by von Arnim et al., who put forward the idea that in contrast to vitamin E, vitamins C and A bear relations to dementia incidence [27]. In the reviewed studies, only one showed a significant correlation with the development of dementia. This 9.6-year follow-up study of 5395 participants older than 55 years found that dietary vitamin E could delay the onset of dementia [26]. Besides the studies listed in Table 1, two others used ω-3 fatty acid supplements (discussed in the next paragraph), both of which demonstrated a significant delay in dementia [29,30]. These analyses also included vitamins A and E in combination with ω-3 fatty acids, although the effects of the vitamins alone were not evaluated.

#### 3.1.2. Vitamin B

Throughout the last century, thiamine (vitamin B1) has been associated with neuro-logical problems, including cognitive deficits and AD, through the manipulation of the brain’s glucose metabolism [31]. Low levels of cobalamin (vitamin B12) are associated with neurocognitive disorders [32]. Biologically, the IL-18 signaling pathway, apoptosis, and AD were identified as significant molecular mechanisms underlying the enhancement of cognitive function by mixed B vitamins [33]. Twelve studies that meet the above criteria have investigated the effectiveness of B-vitamin supplementation in older individuals. All these studies are randomized, placebo-controlled trials, with 2935 participants [34,35,36,37,38,39,40,41,42,43,44,45].

Table 2 summarizes the vitamin B studies. Eleven out of the twelve studies concluded that B vitamins, in general, did not affect cognitive function among non-demented, mild-demented, and demented older persons [34,35,37,38,39,40,41,42,43,44,45], while only one suggested treatment with homocysteine-lowering B vitamins can attenuate the accelerated rate of brain atrophy in older individuals with MCI [36]. However, three studies found that the use of vitamin B supplements could benefit specific subgroups, including subjects with the ins/ins genotype, high plasma ω-3 FA levels, and high plasma DHA levels [34,39,43]. For instance, participants with elevated plasma ω-3 FA levels exhibited reduced rates of cognitive decline and brain atrophy subsequent to B-vitamin supplementation (*p* = 0.023), highlighting the potential benefits of the combined use of ω-3 FAs and B-vitamins [39]. Another post hoc analysis also agrees that ω-3 FAs modified vitamin B efficacy; however, it pointed out that the effectiveness of the B-vitamin supplementation toward cognition is only associated with plasma DHA levels rather than with total ω-3 FA or EPA levels [43].

In terms of cognitive outcomes, two studies measured mental outcomes using the Clinical Dementia Rating Scale Global Score (CDR-global), Clinical Dementia Rating Scale Sum of Boxes (CDR-SOB), memory Z-score, and executive function Z-score, where both showed no significant effect on these measurements [41,43]. Four studies used changes in the rate of brain atrophy as the primary cognitive outcome measure [35,36,39,43]. One of the more extensive pooled post hoc analysis studies that investigated the interaction be-tween aspirin and folic-acid-containing B vitamins in cognitive functioning showed a significant reduction in the whole-brain atrophy rate with supplementation in those without the DHFR 19-bp deletion allele; for example, in the ins/ins genotype [43]. After accounting for aspirin use, the cognitive benefits were more pronounced, and the whole-brain atrophy rate reduction became more significant within the ins/ins genotype participants (*p*-value = 0.004) [43]. Previous studies have indicated that the whole-brain atrophy rate was strongly linked to progression to cognitive decline; a high whole-brain atrophy rate was associated with an increased risk of dementia [46]. A significant reduction in the brain atrophy rate may refer to a decreased risk of dementia in older people with MCI. The pooled data analysis reaffirmed the significance of the whole-brain atrophy rate reduction with B-vitamin consumption prior to decreased serum homocysteine levels in non-aspirin-using older subjects with MCI, reinforcing that homocysteine may contribute to AD [47]. Similarly, another study demonstrated no effect of homocysteine-lowering B vitamins on cognitive decline after a two-year treatment. However, depression symptoms improved, as evidenced by significantly lower Hamilton Depression Rating Scale (HDRS) scores at month 12 [41]. Both studies pointed out the negative interaction effect on the concomitant use of aspirin and B vitamins in cognitive functioning, with *p*-values of 0.009 and 0.005, respectively [43,44].

The mental outcomes in most studies were obtained based on an evaluation of the scaling and scoring system. Compared with the exams that assess cognitive function and symptoms, which are subjective and can be influenced by various factors, magnetic resonance imaging (MRI) offers a more precise and quantifiable assessment of brain damage by providing direct, objective measurements of the brain structure and changes. Atrophy, for example, is indicative of neurodegenerative processes. However, heterogeneity in the results was observed between two studies that administered the same intervention to participants over the same duration. Both studies enrolled MCI older adults and randomly assigned them to either the treatment groups by giving 0.8 mg of folic acid, 20 mg of vitamin B6, and 0.5 mg of vitamin B12 daily or to the placebo groups for 24 months [36,39]. The whole-brain atrophy rates were measured based on MRI scans in these studies. One study found that the average brain atrophy rate per year was 0.76% in the treatment group and 1.08% in the placebo group (*p* = 0.001), indicating a reduction in the rate of brain atrophy of about 30% [36]. High doses of B-vitamin supplementation were concluded to help to slow the progression to AD in older individuals with MCI [36]. Nevertheless, the other study suggested the benefit of B-vitamin supplementation was only observed in subjects with high plasma concentrations of ω-3 fatty acids, meaning their EPA and DHA levels were higher than 590 μmol/L [39]. A similar beneficial effect was not seen in subjects with low ω-3 fatty acid concentrations (<390 μmol/L), emphasizing the need for subgroup identification [39]. This conclusion is further supported by another study that explores the impact of B-vitamin supplementation based on tertiles of baseline plasma ω-3 FA concentrations, as well as separately for EPA and DHA [34]. The results showed that the treatment effect on global cognition was prominent in the older population in the high-DHA tertile compared with the middle tertile (*p* = 0.03). In contrast, no significant interaction was revealed between B vitamins and EPA or the total plasma ω-3 FA level [34].

The discrepancies are most likely because of variants in cognitive outcome measurements, statistical analysis models, the baseline mental status, and the inclusion of a few subjects that contributed to the sampling error; they cannot account for the heterogeneity among all the older populations. In addition, most studies intervened from 12 to 24 months; these durations are relatively short in recognizing cognitive deterioration.

#### 3.1.3. Vitamin D3 and Calcium

Numerous observational studies have established that serum vitamin D levels tend to be significantly lower in individuals with cognitive impairment compared with those among healthy adults [48]. Emerging evidence has highlighted vitamin D’s antioxidant and anti-inflammatory properties, which can protect against neurodegeneration in the brain and reduce AD hallmarks [49].

Our literature search identified seven studies: Five were randomized, double-blind, placebo-controlled trials, and two were observational, prospective, longitudinal cohorts [50,51,52,53,54,55,56], shown in Table 3. Four studies that included n = 3010 participants reported that vitamin D supplementation is associated with cognitive decline in older subjects [50,54,55,56]. Two studies in China provided MCI and AD participants, respectively, with 800 IU of cholecalciferol (vitamin D3) daily for 12 months [50,54]. Improvement of cognitive function in older adults with MCI was achieved by reducing oxidative stress, and amyloid beta (Aβ)-related biomarkers decreased over time in adults with AD, given the accumulation of Aβ protein in the brain is a significant cause of AD, as it results in neuronal dysfunction and death [50,54].

Two longitudinal observational studies followed participants for 5.8–6 years to examine the development of dementia over time [55,56]. One investigated the correlation between dietary intake and cognitive decline by assessing the food frequency questionnaire (FFQ) and neuropsychological testing [55]. The results showed that the total vitamin D intake levels differed by factors such as sex, race, income, educational background, smoking status, and health conditions. Those with a higher total vitamin D intake from food sources tended to be non-blacks, more educated, and non-smokers and had a healthier overall diet [55]. Over a follow-up period of 5.8 years, 329 (18.7%) participants developed dementia; 285 (86.6%) of them were further diagnosed with AD [55]. Participants in the higher tertile had a decreased risk of dementia compared with those in the lower tertile, in which participants did not meet the estimated average requirement (EAR) of vitamin D (*p* = 0.042, hazard ratio (HR) 0.76) [55]. In 2010, a study examined serum 25-hydroxyvitamin D (25(OH)D) levels and dementia and found that participants with severe 25(OH)D deficiency experienced an additional decline of 0.3 Mini-Mental State Examination (MMSE) points per year compared to those with sufficient levels of 25(OH)D [56]. Both studies underlined the importance of vitamin D intake as a preventative strategy against cognitive decline in persons over 65. This finding aligns with the conclusion of another systematic review and meta-analysis that low vitamin D levels were associated with increased dementia risk [57]. One important consideration is that individuals with darker skin colors might be one of the reasons for a low vitamin D status because of the increased melanin content [58]. Given that the majority of those who were included in this systematic review were white [57], findings from Zhao et al. added to this literature by including a diverse and multiethnic cohort (29.9% white, 32% black, and 38.1% other), which increased the generalization of the findings [55].

Bray et al. conducted a study involving older adults in routine cognitive training, physical exercise, and vitamin D3 supplementation [53]. The intervention lasted only 20 weeks, which was relatively shorter than the treatments in the other studies [53]. The results suggested that physical exercise is effective in modifying the connectivity of the default-mode network (DMN)—a brain region that involves cognitive processes, such as remembering and thinking [53]. Meanwhile, the effects of cognitive training and vitamin D supplements were less noticeable [53].

Two long-term randomized, double-blind, placebo-controlled trials that focused on women only were conducted in the United States [51,52]. One study recruited cognitively normal postmenopausal African American women. It provided the treatment group with sufficient amounts of vitamin D and calcium supplement tablets for three years to achieve a serum 25(OH)D level of >30 ng/mL [51]. The change over time did not differ significantly between the groups [51]. Likewise, another study involved women across all cognitive statuses, who were treated with 1000 mg of calcium carbonate and 400 IU of vitamin D3 daily and followed for 7.8 years [52]. Researchers reported no significant difference in the incidence of cognitive impairment between the placebo and treatment groups [52]. One potential explanation for the insignificant result is that the dose (400 IU) was selected in the early 1990s, which is substantially distinct from the recommended dietary allowance (RDA) nowadays (600 IU for 19 years and older; 800 IU for older adults > 70) [59]. Additionally, the lack of a record on the severity of baseline diseases among the participants or adjustments for medication use can lead to potential confounders. Medications, such as laxatives, steroids (prednisone), seizure medications (phenobarbital and phenytoin), and blood pressure medications (spironolactone and nifedipine), can potentially lower vitamin D levels [60]. Nonetheless, assessments of non-supplementation sources of vitamin D, such as food and sunlight exposure, are confounding factors that are critical to rule out [58].

### 3.2. Polyunsaturated Fatty Acids (PUFAs)

PUFAs can be divided into two primary categories: omega-3 and omega-6 fatty acids (ω-6 FAs) [61]. Several studies have revealed a close association among docosapentaenoic acid (DPA), omega-6 arachidonic acid (AA), and a lower rate of cognitive decline [62,63]. Because of the application of the inclusion criteria, the results obtained from our literature review do not pertain to ω-6 FAs. Thus, we will only discuss findings showing correlations between health outcomes and intakes or biomarkers related to ω-3 FAs. There are three major types of ω-3 FAs: α-linolenic acid (ALA), EPA, and DHA [64], where EPA and DHA belong to long-chain compounds because they contain 20 or more carbon atoms [65]. Among the latter, DHA constitutes up to 60% of the lipids associated with neuronal machinery [66]; most agents derived from EPA and DHA are anti-inflammatory or display antioxidant activity in the hippocampal cortex [67].

Among the reviewed studies investigating the influence of PUFAs on dementia-related outcomes in older adults, nine RCTs and a secondary analysis (including 7609 participants), as shown in Table 4, demonstrated compelling results for a positive relationship between PUFAs and a lower risk of dementia [29,30,68,69,70,71,72,73,74]. Three studies enrolled 101 participants and administered a combination of EPA, DHA, vitamin A, and vitamin E in various ratios, reporting beneficial effects in reducing cognitive decline associated with supplementation [29,30,70]. In the larger cohort that compared the (1) placebo with the ω-3 FA supplement intervention group, (2) multi-domain intervention with the placebo group, and (3) multi-domain with the supplement intervention group, the combination of the multi-domain with supplementation showed significant improvement in the delay in dementia, as determined using the free and cued selective reminding test (FCSRT) and MMSE test at 36 months [68]. This study comparatively lasted longer with a larger sample size. Yet, the nature of a post hoc analysis is subject to bias, given that calculations were performed from a previous study [75]. Two studies included 2759 non-demented older adults with EPA and DHA use [69,71]. Although Maltais et al. is not an RCT, this secondary analysis offers valuable information related to a large sample size and a promising study duration. They found dose–response associations with a lower risk for the development of dementia and serum levels of these FAs [69,71]. High-quality evidence from long-term prospective studies, such as the 17-year observational study, with a diet assessed by blood tests and dietary surveys at every follow-up, strongly supports that higher levels of fish-derived long-chain ω-3 FAs are associated with lower dementia-related outcomes in healthy older adult populations [76]. Controlling for confounders, such as lifestyle and diet quality, improved the preciseness of the results.

Conversely, four studies involving 3266 participants showed no significant effect [72,73,74,77]. These studies used DHA only or a combination of DHA and EPA, as interventions and did not include other vitamins [72,73,74,77]. The combined EPA and DHA doses ranged from 230 mg to 1800 mg in these studies [72,73,74,77]. Moreover, most participants had normal cognitive function at the baseline, with one study not specified [72,73,74,77]. The variation in the results is because of the extensive range of supplement doses, different starting mental states, and the absence of dietary reviews because some people may occasionally eat fish or other foods high in omega-three fatty acids. The Institute of Medicine (IOM) has not established dietary reference intakes (DRIs) for PUFAs specifically. Also, there are insufficient data to establish an EAR, and only adequate intake (AI) recommendations have been made for ALA and not for EPA and DHA [65]. The lack of dietary analysis and the fact that dietary eating patterns changed throughout the trial make it difficult to assess the effect of the PUFA tablets on the diets of older adults. Also, the inclusion of mentally healthier older adults would only serve to minimize the relationships between PUFAs and health status.

### 3.3. Probiotics

Researchers have demonstrated that the gut–brain axis facilitates communication between the gut microbiota and the brain and that bacterial metabolites can have either neuroprotective or neurotoxic effects on the central nervous system (CNS) [78]. This underscores the need for more study of probiotics as a possible adjunct therapy in dementia. However, different probiotic species and strains are used in each study; three out of the four studies (n = 199) that we examined demonstrated a significant positive correlation between probiotic supplementation and reduced cognitive decline, as opposed to a total of four studies (n = 239) [79,80,81,82] (see Table 5). All three studies focused on older adults with MCI at the baseline and administered approximately 1–2 × 10^10^ CFU of probiotics for 12 to 24 weeks [79,81,82]—one employed neuroimaging and quantitative techniques to examine gray matter atrophy [82]. The new findings in neuroimaging research allow for doctors to study the brain structure at an outstanding level of detail. Therefore, this method has become a very precise and reliable tool to diagnose dementia and to observe its progress. This type of technology complements the usual dementia-rating tests and, combined with these approaches, offers a more personal and in-depth examination. The study’s positive findings on *B. breve A1* are further supported by Xiao et al., who administered 2 × 10^10^ CFU of *B. breve A1* to 80 adults over 60 years old, with MCI, for 16 weeks, resulting in improved memory [83]. In contrast, Hsu et al. studied 40 AD subjects and treated them for 12 weeks with a multi-strain probiotic regimen [80]. Although the study showed a tendency for less cognitive decline, it did not reach statistical significance [80]. This finding is supported by a study with a larger sample size that examined the effect of probiotics on physically active older adults, which implies that taking probiotic supplements does not significantly improve cognitive abilities in those who have engaged in physical activity [84]. That is inconsistent with another multi-strain-probiotic study, which showed improved cognitive function and sleep quality [79]. However, the heterogeneity among studies makes it difficult to interpret the results because of the differences in subject demographics, probiotic species and strains used, and length of the intervention, which could all contribute to conflicting results. But with study periods lasting from 12 to 23 weeks, much longer interventions are needed.

## 4. Discussion

MCI and AD have emerged as major public health issues, driven in part by longer lifespans and the growing number of individuals experiencing chronic health conditions as they age [85]. As of now, no cure has been discovered for treating MCI or AD. Consequently, it is crucial to explore methods for reducing the risk of cognitive impairment with the goal of slowing its progression. Dementia is more common as people grow older; evidence suggests that although it is not a normal part of aging, about one-third of all people aged 85 or older have some form of dementia [86]. Although numerous studies in this area have been published, there is a lack of investigation into the impact of these supplements on cognitive impairment among older adults. Hence, the current evidence from observational studies and interventional trials investigating specific micronutrients in relation to cognitive decline and dementia in older persons aged at least 65 years was reviewed.

Emerging evidence indicates that supplements, such as vitamin D, PUFAs, and probiotics, are linked to reduced cognitive decline and a lower risk of dementia. In contrast, vitamins A, B, C, and E generally show no significant effects. However, variability in study methodologies—primarily RCTs along with some longitudinal observational studies—complicates direct comparisons. Consequently, the general conclusions are based on the available data and discussed accordingly.

Positive outcomes have been observed in older individuals using vitamin D, PUFAs, and probiotics. Notably, among the five studies on vitamin D, three are RCTs, while two are prospective longitudinal cohort studies with durations of five years or more. Although both cohort studies indicated a positive relationship, three of the five RCTs did not support this connection. Additionally, the highest dose of vitamin D among these studies was 10,000 IU, while the recommended dose for 51-year-old or older adults is from 600 to 800 IU or achieving a serum level of 20 ng/mL [87]. Only one study focused on assessing serum vitamin D levels over the course of three years and varied the dosage to achieve the targeted 30 ng/mL concentration [51]. That study, which had a long duration of nearly eight years and the largest sample size of over 4000 participants, focused explicitly on women’s health and found no effect from daily supplementation with 400 IU of vitamin D3 [52]. This finding is consistent with a prior systematic review and meta-analysis that examined nine RCTs involving 2345 participants [88]. Of the nine studies we reviewed on PUFAs, eight are RCTs, and one is a cohort study. Five of these studies demonstrated significant associations between DHA and EPA and reduced cognitive decline risk. However, a recent systematic review published in 2020 found little to no effect of PUFAs on neurocognitive outcomes or cognitive impairment [89]. Probiotics have yielded more promising results; three studies we reviewed indicate a connection between microbiota and cognitive improvement. This finding is consistent with conclusions from two other literature reviews examining gut microbiota and dementia in older adults, which indicate that probiotic consumption positively impacts AD [90,91].

Most studies we found showed that vitamin A, B, C, and E supplementation is not significantly related to a lower risk of dementia. Despite a comprehensive database search, no studies related to vitamins A, C, and E met the inclusion criteria. However, we investigated five studies on the relationship between antioxidants and dementia, considering the essential role antioxidants are believed to have in dementia pathogenesis. These trials did not show a beneficial effect of antioxidant supplementation; however, some studies highlight the potential impacts of dietary vitamin E intake and serum levels of vitamins A and C on MCI and AD. Further studies are required to ascertain the effect of antioxidant supplementation on the delay in the onset of dementia among adults aged 65 and above.

Out of the twelve trials to prove the efficiency of vitamin B supplementation in cognitive decline, only one was positive. All these studies used different forms of vitamin B, including folic acid, vitamin B3, vitamin B6, and vitamin B12, in various ratios. The only valuable trial study used a combination of 0.8 mg of folic acid, 0.5 mg of vitamin B12, and 20 mg of vitamin B6 daily performed alone [36]. The successful trial suggested that positive results were observed only in subjects with high plasma levels of ω-3 FAs [39]. Similarly, another study indicated that the efficacy of vitamin B supplementation is dependent on individual factors, particularly in non-aspirin users with the ins/ins genotype [43]. Our findings are at odds with a meta-analysis that reviewed 95 studies involving 46,175 participants. The meta-analysis underlined that supplementation with B vitamins is related to reduced cognitive decline. Moreover, that study showed that dietary folate intake, but not those of vitamins B12 or B6, is inversely related to the risk for dementia in the older population [92].

**Figure 2:**
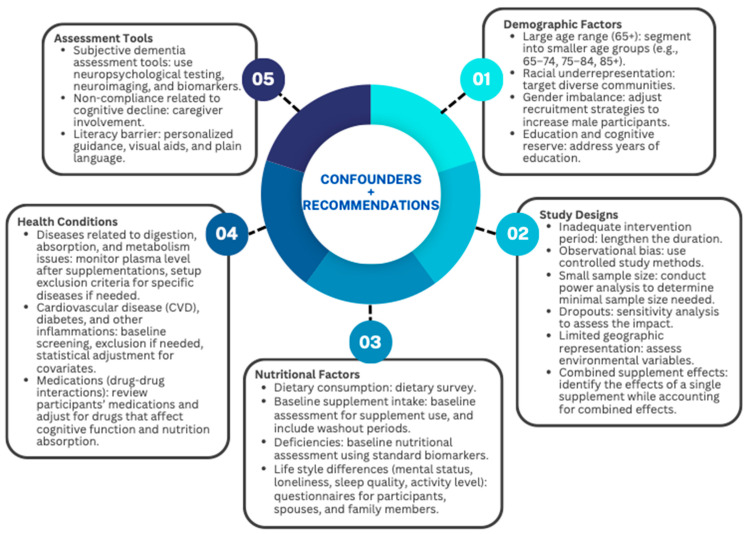
Summary of the major experimental confounders and recommendations.

Detailed consideration of the main confounders identified in our review, with guidance on appropriate methods to address them, is presented in Figure 2. Confounders are categorized into five domains, including demographics, study designs, nutritional status, health conditions, and assessment tools. The studies included in our review involved participants across a wide age range, from 65 and older. As research has shown that older adults are not a homogenous group, with significant physiological differences between those in their 60s and their 80s, we suggest researchers consider dividing the older adult group into at least three categories: an early-old age group (65–74), a mid-old age group (75–84), and a late-old age group (85). This will allow for the examination of the true effectiveness and potential intervention effect modifiers known to be present at different stages of aging. Although it would be unreasonable to expect an equal number of individuals to be equally distributed across three age groups, doing so can significantly help address age-related questions. Furthermore, the studies identified in this review recruited predominately Caucasian participants and had a higher ratio of female subjects. To improve population representativeness, other studies should aim to recruit among more diverse communities or adjust for ethnicity and gender effects in relevant analyses. Although it may not be possible to achieve perfect matching in interventional studies, researchers should still collect baseline education years, as higher educational attainment and cognitive reserve relate negatively to dementia risk and may delay the onset of symptoms.

As for the study design, longitudinal observational studies should include a minimum follow-up period of ten years to adequately capture long-term effects. In RCTs, dosage regimens should be customized based on current guidelines and individual patient profiles to achieve the optimal results, given the lack of a universal dosage applicable to all individuals. Another critical point that should be made aware is that outcome measures differ. For example, RCTs and cross-sectional studies used cognitive performance as outcome measures, and longitudinal studies used cognitive decline or risk of AD or dementia. Comparisons across studies are also hampered by different methodologies for measuring and reporting cognitive outcomes. The follow-up period ranged from 6 to 20 years for longitudinal cohort studies, and interventional studies lasted from 12 weeks to 3 years. Cohort study designs can infer associations and temporal correlations but are not confirmatory of causality. Some trials have shorter follow-up periods, which could underestimate the full long-term intervention effects. Although extended follow-up is essential in finding changes in cognitive functioning, the optimal duration and time of the extended follow-up are yet to be known. Nevertheless, because of a paucity of complete human data from both observational and interventional studies, the ability is limited in drawing clear clinical value from these supplements. Power analysis is to be done to determine the minimum sample sizes appropriate for deduction. Another equally important aspect of the interpretation of the results is that if a participant deceases at some point during the follow-up period, that person will no longer develop dementia. That is, if nutritional exposure has a raised risk of mortality then the observed number of cases of diagnosed dementia may represent an underestimate compared with the actual value. Scanning for interfering environmental effects is essential, especially when one is dealing with nutritional supplementation, for example, vitamin D and sun exposure. Environmental exposures may form confounder variables in addition to the action of other supplements, whether those are multi-vitamins taken by participants or provided by the researchers and meant for the analysis of synergism or combined effects.

To identify the effect of supplementation only, several studies assessed dietary intake to exclude possible interference from food sources. This is important because some people may already have adequate intakes of these micronutrients in their diet, and supplementation will only have a total absorption rate in the presence of a pre-existing deficiency. Lifestyle factors, such as participants’ physical activity levels, sleep quality, social status, pre-existing depression, and stress levels, may also influence outcomes. Thus, tracking this information is essential, and support from spouses and family members is recommended and desired. Past medical history (PMH), mostly in the areas of metabolic, cardiovascular, immune, or inflammatory disorders, must be taken into consideration. Without proper gastrointestinal tract health and function, the absorption of supplements will be impaired, and diseases, such as diabetes, stroke, heart failure, hypertension, and obesity, increase the risk for developing dementia. Moreover, it is important that up-to-date medications are reviewed to identify any potential drug interactions that supplements may have on the participants’ currently prescribed medications. Adjustments can also be made for drugs known to interfere with normal cognitive functions.

Cognitive outcomes are often measured as a part of the studies we reviewed through the MMSE [93], the Diagnostic and Statistical Manual of Mental Disorders (DSM) [93], and the CDR-SOB scoring systems [94]. Although the MMSE is generally accepted as a tool for assessing cognitive function, it has some limitations in terms of vulnerability to influence by non-cognitive factors, such as educational level and lack of sensitivity to subtle signs of early dementia. Some studies also include subjective brain-imaging techniques to obtain the atrophy rates in the brain more objectively and precisely, as scoring systems are prone to interpretative variability. Methodological issues related to cognitive outcome measurements should be addressed with more sensitive diagnostic tools. For example, although cognitive assessments can provide specific insights, MRI imaging may offer superior sensitivity for detecting subtle changes. Furthermore, cognitive assessment tools administered with the help of primary caregivers can improve the validity and reliability of outcome measurements. Even further, caregiver assistance can help to track and manage compliance rates associated with cognitive decline and other similar issues. To sum up, future research should focus on interventions that investigate relations among micronutrient supplementation, dietary intake, plasma micronutrient levels, and cognitive outcomes. Of specific importance is targeting the age cohort between 65 and 85 years because this group is exposed to higher potential for cognitive decline.

The main limitation of our systematic review is that we only compiled research on populations in the 65-and-over age group with variable baseline mental statuses (non-demented, MCI, and demented). We did not perform any analyses for smaller age intervals or cases with a specific baseline cognitive status. The number of research papers that separated results by both age and cognitive status was low and would require larger databases with more detailed age results than provided in these studies. Age distinctness is currently not being widely provided by the authors, and we, therefore, omitted this analysis. The strength of the present systematic review is also limited, as we only included manuscripts published between 2004 and 2024. This is a justifiable time limitation to include relevant study results. However, it also means that many studies published before this time frame and relevant information are not considered. One aspect to mention is that many of these studies on supplements are limited to specific micronutrients. It is beyond the scope of this review to survey the evidence for all macronutrients, micronutrients, minerals, and other forms of vitamins. We selected, for review, specific vitamins and minerals that may be more implicated in dementia progression, omitting, for example, the subgroup of B vitamins or antioxidants other than vitamins A, C, and E, as well as macronutrients and herbal supplements. Lastly, as most studies on probiotics we explored dealt with multi-strain probiotics, it was not possible for us to determine whether single-strain probiotics might have produced similar cognitive effects with the study population. Conclusions cannot be reached, given that single-strain probiotic studies in the elderly population are less common and that comparative data were even less available.

## 5. Conclusions

This review has provided a strong body of evidence to explain the nature of the contribution of micronutrient supplementation in modulating the progressions of MCI and AD. Supplementations of vitamin D, probiotics, and PUFAs, particularly ω-3 FAs, tend to be beneficial in delaying the process of cognitive decline and attenuating the risk for dementia. On the contrary, vitamins A, B, C, and E are not promising enough in this regard.

The history of dementia in families—independently of known genetic risks, the latter being a recognized risk factor—should also be considered. Additionally, gender differences may influence study outcomes. Evidence-based advice with treatment and disease prevention strategies should be given to those older adults suffering from pre-dementia conditions to develop effective, cost-effective, and sustainable solutions. This becomes especially relevant as, up to now, there are no curative treatments for such conditions.

## Figures and Tables

**Table 1:** Description of included studies—vitamins A, C, and E.

Author, Publication Year	Sample Size	Baseline	Study Design	Age Range	Intervention	Length	Cognitive Outcome	Main Result
Fillenbaum et al., 2005 [27]	616	Non-demented and AD	Secondary analysis	72.9 ± 2	NA—self-reported vitamin C and E supplement use	Long term (15 years)	SPMSQ scores ^1^; DSM ^2^; MMSE ^3^	=for VC; VE
von Arnim et al., 2012 [26]	74	MCI	Observational—Secondary analysis	80 ± 9	NA—serum antioxidant measure (including vitamin C, vitamin E, β-carotene, lycopene, and coenzyme Q10)	Cross-sectional	MMSE ^3^; semantic word fluency; verbal memory; visual memory	=for VE ↑ for VC; VA
Kryscio et al., 2017 [24]	3786	Non-demented, MCI, and AD	Randomized double blind placebo controlled	67.6 ± 5.3 (>60 y.o.)	(1) vitamin E (400 IU/day); (2) selenium (200 μg/day); (3) vitamin E (400 IU/day) + selenium (200 μg/day)	Long term (6 years)	MIS ^4^; TICS-m ^5^	=for VE; selenium (men-only trial)
Devore et al., 2011 [30]	5395	Non-demented	Prospective cohort	67.6 ± 0.5 (>55 y.o.)	NA—dietary antioxidant assessment	Long term (9.6 years)	MMSE ^3^; GMS ^6^; DSM ^2^; NINCDS-ADRDA ^7^	↑ (in subjects with higher dietary vitamin E consumption)
Koch et al., 2021 [25]	996	Non-demented, MCI, and AD	Case cohort	79 ± 2	NA—serum antioxidants	Long term (5.9 years)	3MS ^8^; CDR ^9^; ADAS ^10^	=for VA; VE

^1^ Short Portable Mental Status Questionnaire; ^2^ Diagnostic and Statistical Manual of Mental Disorders; ^3^ Mini-Mental State Examination; ^4^ Memory Impairment Screen; ^5^ Telephone Interview for Cognitive Status; ^6^ Geriatric Mental State Schedule; ^7^ National Institute of Neurological and Communicative Disorders and Stroke and the Alzheimer’s Disease and Related Disorders Association; ^8^ Modified Mini-Mental State Examination; ^9^ Clinical Dementia Rating Scale; ^10^ Alzheimer’s Disease Assessment Scale. + indicates combination of two nutrients; = indicates equal; ↑ = increased.

**Table 2:** Description of included studies—vitamin B.

Author, Publication Year	Sample Size	Baseline	Study Design	Age Range	Intervention	Length	Cognitive Outcome	Main Result
Ford et al., 2010 [44]	299	Non-demented	Randomized; double blind; placebo controlled	79 ± 2.8	(1) 400 μg of B12 + 2 mg of folic acid + 25 mg of B6 once daily	Medium-term intervention (24 months); Long-term FU (8 years)	TICS ^1^; MMSE ^2^; BDI ^3^	=
Kwok et al., 2020 [43]	279	MCI	Randomized; placebo controlled	78 ± 5.4	(1) 500 μg of methylcobalamin + 400 μg of folic acid once daily	Medium term (24 months)	CDR-SOB ^4^; CDR-global ^5^; memory Z-score; executive function Z-score; HDRS ^6^	=
Wu et al., 2021 [42]	545	MCI	Randomized; placebo controlled; pooled (UK and HK)	77.1 ± 5.3	(1) UK: 800 μg of FA + 500 μg of cyanocobalamin + 20 mg of vitamin B6 (2) HK: 400 μg of FA + 500 μg of methylcobalamin	Medium term (24 months)	CDR-global ^5^; CDR-SOB ^4^; memory Z-score; executive Z-score, brain atrophy rate	= (+ only in non-aspirin users specifically with the ins/ins genotype)
McMahon et al., 2006 [41]	276	Non-demented	Randomized; double blind; placebo controlled	73.5 ± 5.8	(1) 1000 μg of folate + 500 μg of vitamin B12 + 10 mg of vitamin B6	Medium term (24 months)	MMSE ^2^; RAVLT ^7^; WMS ^8^; COWAT ^9^; CFT ^10^	=
Kwok et al., 2011 [40]	140	MCI	Randomized; double blind; placebo controlled	78.2 ± 7.9	(1) 1 mg of methylcobalamin + 5 mg of folic acid daily	Medium term (24 months)	MDRS ^11^; MMSE ^2^; CSSD ^12^	=
Aisen et al., 2008 [39]	409	AD	Randomized; double blind; controlled	76.3 ± 8	(1) 5 mg of folate + 25 mg of vitamin B6 + 1 mg of vitamin B12 daily	Medium term (18 months)	MMSE ^2^; CDR-SOB ^4^; ADCS-ADL ^12^; Neuropsychiatric inventory; Quality of life–AD	=
Jernerén et al., 2015 [38]	168	MCI	Randomized; placebo controlled	76.6 ± 0.7	(1) 0.8 mg of folic acid + 20 mg of vitamin B6 + 0.5 mg of vitamin B12 daily	Medium term (24 months)	Brain atrophy rates	= (+ only in subjects with high plasma ω-3 fatty acid levels)
Uffelen et al., 2008 [37]	152	MCI + AD	Randomized; placebo controlled	75 ± 2.9	(1) 5 mg of folic acid + 0.4 mg of vitamin B12 + 50 mg of vitamin B6 daily + moderate-intensity walking twice weekly	Medium term (12 months)	MMSE ^2^; AVLT ^13^; VFT ^14^; DSST ^15^; SCWT-A ^16^	=
Stott et al., 2005 [36]	185	Non-demented	Randomized; double blind; placebo controlled	74 ± 8	(1) 2.5 mg of folic acid + 500 μg of vitamin B12 + 25 mg of vitamin B6 + 25 mg of riboflavin daily	Medium term (12 months)	MMSE ^2^; DSST ^15^	=
Smith et al., 2010 [35]	271	MCI	Randomized; double blind; placebo controlled	76.6 ± 5.2	(1) 0.8 mg of folic acid + 0.5 mg of vitamin B12 + 20 mg of vitamin B6 daily	Medium term (24 months)	MMSE ^2^; CAMDEX ^16^, TICS-M ^1^; Brain atrophy rates; GDS ^17^	↑
Orr et al., 2023 [34]	20	MCI	Randomized; double blind; placebo controlled	76.2 ± 9.9	(1) nicotinamide riboside, 250 mg twice a day	Short term (10 weeks)	MoCA ^18^; EXIT ^19^; CLOX ^20^; GDS ^17^; GAS ^21^; Brain atrophy rates	=
van Soest et al., 2022 [33]	191	Non-demented	Randomized; double blind; placebo controlled	71.6 ± 5.9	(1) 400 µg of folic acid + 500 µg of vitamin B12 daily	Medium term (24 months)	RAVLT ^7^; Digit span task; TMT ^22^; SCWT ^16^; SDMT ^23^; Letter fluency	= (+ only in subjects with higher plasma DHA levels)

^1^ Telephone Interview for Cognitive Status; ^2^ Mini-Mental State Examination; ^3^ Beck Depression Inventory; ^4^ Clinical Dementia Rating Scale Sum of Box Scores; ^5^ Clinical Dementia Rating Scale Global; ^6^ Hamilton Depression Rating Scale; ^7^ Rey Auditory Verbal Learning Test; ^8^ Wechsler Memory Scales; ^9^ Controlled Oral Word Association Test; ^10^ Category Word Fluency Test; ^11^ Montgomery–Asberg Depression Rating Scale; ^12^ Cornell Scale for Depression in Dementia; ^13^ Verbal Fluency Test; ^14^ Digit Symbol Substitution Test; ^15^ Stroop Color and Word Test; ^16^ Comprehensive Assessment for Dementia in People with Down Syndrome; ^17^ Global Deterioration Scale; ^18^ Montreal Cognitive Assessment; ^19^ Executive Interview; ^20^ Clock Drawing Task; ^21^ Goal Attainment Scaling; ^22^ Trail-Making Test; ^23^ Symbol Digit Modalities Test. + indicates combination of two nutrients; = indicates equal; ↑ = increased.

**Table 3:** Description of included studies—vitamin D3 and calcium.

Author, Publication Year	Sample Size	Baseline	Study Design	Age Range	Intervention	Length	Cognitive Outcome	Main Result
Jia et al., 2019 [49]	210	AD	Randomized; double blind; placebo controlled	67.78 ± 5.19	(1) 800 IU of vitamin D3 daily	Medium term (12 months)	WAIS-RC ^1^; ADL ^2^; IQ score; MMSE ^3^	↑
Owusu et al., 2019 [50]	260	Non-demented	Randomized; double blind; placebo controlled	68.95 ± 3.5	(1) 2400 IU, 3600 IU, or 4800 IU of vitamin D3 with 1200 mg of Ca to achieve a serum level of >30 ng/mL; (2) Placebo with 1200 mg of Ca daily	Long term (3 years)	MMSE ^3^; orientation to place; attention to calculation; recall; naming; repetition; comprehension; reading; writing; drawing	= (specifically in older African American women)
Rossom et al., 2012 [51]	4152	MCI; AD; non-demented	Randomized; double blind; placebo controlled	70.8 ± 5.8	(1) 1000 mg of calcium carbonate and 400 IU of vitamin D3 daily	Long term (7.8 years)	DSM-IV ^12^; CERAD ^4^; Burnam score; digits forward and backward; PMA vocabulary; card rotations; letter and category fluency; California Verbal Learning Test; Benton Visual Retention Test; finger tapping	= (specifically in women)
Bray et al., 2023 [52]	120	MCI	Randomized; double blind; placebo controlled	73.89 ± 6.5	(1) 60 min of physical exercise; (2) 30 min of cognitive training + 60 min of physical exercise; (3) 60 min of physical exercise + vitamin D3 (10,000 IU/pill) 3 times per week; (4) 30 min of cognitive training + 60 min of physical exercise + vitamin D3 3x/week	Short term (20 weeks)	ADAS-Cog-13 ^5^; TMT ^6^; FBC ^7^	= (↑ if with physical exercise)
Yang et al., 2020 [53]	183	MCI	Randomized; double blind; placebo controlled	66.9 ± 6.1	(1) 800 IU of vitamin D3 daily	Medium term (12 months)	WAIS-RC ^8^; FSIQ ^9^; MMSE ^3^	↑
Zhao et al., 2020 [54]	1759	Non-demented	Prospective longitudinal cohort	76.9 ± 6.5	NA	Long term (5.8 years)	DSM ^10^; NINCDS-ADRDA ^11^	↑
Llewelly et al., 2010 [55]	858	MCI; AD; non-demented	Prospective longitudinal cohort	73.95 ± 7.9	NA	Long term (6 years)	MMSE ^3^; TMT A and B ^6^	↑

^1^ Wechsler Adult Intelligence Scale Revised; ^2^ Activity of Daily Living; ^3^ Mini-Mental State Examination; ^4^ Consortium to Establish a Registry for Alzheimer’s Disease; ^5^ Alzheimer’s Disease Assessment Scale Cog 13; ^6^ Trail-Making Test; ^7^ Functional Brain Connectivity; ^8^ Wechsler Adult Intelligence Scale Revised; ^9^ Full-Scale Intelligence Quotient; ^10^ Diagnostic and Statistical Manual of Mental Disorders; ^11^ National Institute of Neurological and Communicative Disorders and Stroke–Alzheimer’s Disease and Related Disorders Association; ^12^ Diagnostic and Statistical Manual of Mental Disorders. + indicates combination of two nutrients; = indicates equal; ↑ = increased.

**Table 4:** Description of included studies—*PUFAs*.

Author, Publication Year	Sample Size	Baseline	Study Design	Age Range	Intervention	Length	Cognitive Outcome	Main Result
Power et al., 2022 [28]	30	Non-demented	Randomized; double blind; placebo controlled	69.03 ± 4.41	(1) 1 g of fish oil (contains 430 mg of DHA and 90 mg of EPA) + 22 mg of carotenoids (10 mg of lutein, 10 mg of meso-zeaxanthin, and 2 mg of zeaxanthin) + 15 mg of vitamin E daily	Medium term (24 months)	MoCA ^1^; RBANS ^2^; CANTAB ^3^	↑ (with ω-3FAs, xanthophyll, carotenoids, and vitamin E)
Chhetri et al., 2018 [67]	1293	MCI	Randomized; double blind; placebo controlled	75.39 ± 4.32	(1) 800 mg of DHA+ 225 mg of EPA; (2) multi-domain (nutritional counseling, physical exercise, and cognitive stimulation) + placebo; (3) multi-domain + 800 mg of DHA+ 225 mg of EPA	Long term (3 years)	FCSRT ^4^; MMSE ^5^; WAIS-R ^6^; CNT ^7^; TMT ^8^; COWAT ^9^; DSST ^10^	↑
Giudici et al., 2020 [73]	1445	Non-demented	Randomized; placebo controlled	75.3 ± 4.4	(1) Multi-domain intervention (includes cognitive stimulation + physical activity + nutritional counseling) + 400 mg of DHA + ≤112.5 mg of EPA twice daily; (2) Multi-domain intervention + placebo; (3) 400 mg of DHA + ≤112.5 mg of EPA twice daily	Long term (3 years)	MMSE ^5^; DSST ^10^; FCSRT ^11^; CNT ^7^	=
* Maltais et al., 2022 [68]	1680	Non-demented	Secondary data analysis	75.9 ± 4.7	(1) 400 mg of DHA + 112.5 mg of EPA twice daily	Long term (5 years)	FCSRT ^11^; MMSE ^5^; DSST ^10^; WAIS ^12^; CNT ^7^; COWAT ^9^; TMT ^8^; CDR score ^13^	↑ (Subjects with high Hcy levels benefit less)
Tabue-Tegu et al., 2018 [74]	1464	Non-demented	Randomized; placebo controlled	75.37 ± 4.62	(1) 800 mg of DHA; (2) Multi-domain intervention (includes nutrition, physical activity, and cognition)	Long term (3 years)	MMSE ^5^; TMT ^8^; FCSRT ^11^; DSST ^10^; CNT ^7^; COWAT ^9^	=
Rondanelli et al., 2012 [69]	25	MCI	Randomized; placebo controlled	86 ± 6	(1) DHA (720 mg) + EPA (286 mg) + vitamin E (16 mg) + soy phospholipids (160 mg) + tryptophan (95 mg) + melatonin (5 mg)	Short term (12 weeks)	MMSE ^5^; Short-term memory; Long-term memory; Attentional abilities; Visuo-constructional and visuo-spatial abilities	↑
Daďová et al., 2022 [75]	55	Not specified	Randomized; placebo controlled	70.9 ± 3.9	(1) 105 mg of DHA + 125 mg of EPA + exercise training; (2) placebo + exercise training	Short term (16 weeks)	POBAV ^14^; BDNF ^15^	= (women only; ↑ in physically active subjects)
Van de Rest et al., 2008 [76]	302	Non-demented	Randomized; double blind; placebo controlled	69.7 ± 3.4	(1) 400 mg of EPA + DHA; (2) 1800 mg of EPA + DHA	Medium term (6 months)	Word-Learning Test; DGS ^16^; TMT ^8^; SCWT ^17^; VFT ^18^	=
Thomas et al., 2020 [70]	1279	Non-demented	Prospective longitudinal cohort	74.3 ± 4.9	NA—examined plasma EPA and DHA	Long term (17 years)	CDR^13^-global; MMSE ^5^; TMT-A ^8^; MRI ^19^	↑
Stavrinou et al., 2020 [29]	46	MCI	Randomized; double blind; placebo controlled	78.8 ± 7.3	(1) 810 mg of EPA + 4140 mg of DHA + 1800 mg of gamma-linolenic acid + 3150 mg of linoleic acid + 0.6 mg of vitamin A, 22 mg of vitamin E + 760 mg of pure γ-tocopherol	Medium term (6 months)	ACE-R ^20^; MMSE ^5^; STROOP ^21^	↑

* Maltais et al. is not an RCT but provides valuable insights. ^1^ Montreal Cognitive Assessment; ^2^ Assessment of Neuropsychological Status; ^3^ Cambridge Neuropsychological Test Automated Battery; ^4^ Free and Cued Selective Reminding Test; ^5^ Mini-Mental State Examination; ^6^ Wechsler Adult Intelligence Scale; ^7^ Computerized Neurocognitive Test; ^8^ Trail-Making Test; ^9^ Controlled Oral Word Association Test; ^10^ Digit Symbol Substitution Test; ^11^ Free and Cued Selective Reminding Test; ^12^ Wechsler Adult Intelligence Scale; ^13^ Clinical Dementia Rating Score; ^14^ Test Pojmenování OBrázků a jejich Vybavení; ^15^ Brain-Derived Neurotrophic Factor; ^16^ Wechsler Digit Span Task; ^17^ Stroop Color–Word Test; ^18^ Verbal Fluency Test; ^19^ Magnetic Resonance Imaging; ^20^ Addenbrooke’s Cognitive Examination Revised; ^21^ Stroop Color and Word Test. + indicates combination of two nutrients; = indicates equal; ↑ = increased.

**Table 5:** Description of included studies—*Probiotics*.

Author, Publication Year	Sample Size	Baseline	Study Design	Age Range	Intervention	Length	Cognitive Outcome	Main Result
Fei et al., 2023 [78]	42	MCI	Randomized; placebo controlled	76.40 ± 9.61	(1) 2 g (>2 × 10^10^ CFU/g) of probiotics daily (includes *L. plantarum*, *L. rhamnosus*, *L. johnsonii*, *L. paracasei*, *L. salivarius*, *L. acidophilus*, *L. casei*, *L. reuteri*, *L. fermentum*, *L. lactis*, *Bifido. lactis*, *Bifido. animalis*, and *Bifido. infantis*)	Short term (12 weeks)	MMSE ^1^; MoCA scores ^2^; PSQI ^3^	↑
Hsu et al., 2023 [79]	40	AD	Randomized; double blind; controlled	75.8 ± 7.3	(1) 1 × 10^10^ CFU/capsule of probiotics daily (*Bifidobacterium longum* subsp. *infantis* BLI-02, *B. breve* Bv-889, *B. animalis* subsp. *lactis* CP-9, *B. bifidum* VDD088, and *Lactobacillus plantarum* PL-02)	Short term (12 weeks)	MMSE ^1^; CDR ^4^; ADAS-Cog ^5^; ADL test ^6^	=
Kobayashi et al., 2019 [80]	27	MCI	Open-label, single-arm study	83.2 ± 4.9	(1) >1 × 10^10^ CFU/capsule of *B. breve* A1 daily	Short term (24 weeks)	MMSE ^1^; DSST ^7^; POMS2 ^8^; TMD ^9^	↑
Asaoka et al., 2022 [81]	130	MCI	Randomized; double blind; placebo controlled	78 ± 6.1	(1) *B. breve* MCC1274 (contains 2 × 10^10^ CFU)	Short term (24 weeks)	ADAS-Cog ^5^; MMSE ^1^; brain atrophy rates; VSRAD scores ^10^	↑

Lactobacillus = L.; Bifidobacterium = Bifido. ^1^ Mini-Mental State Examination; ^2^ Montreal Cognitive Assessment Scale; ^3^ Pittsburgh Sleep Quality Index; ^4^ Clinical Dementia Rating Score; ^5^ Alzheimer’s Disease Assessment Scale Cog; ^6^ Activities of Daily Living; ^7^ Digit Symbol Substitution Test; ^8^ Profile of Mood States, 2nd Edition; ^9^ Total Mood Disturbance; ^10^ Voxel-based Specific Regional Analysis System for Alzheimer’s Disease. = indicates equal; ↑ = increased.

## Data Availability

No new datawere created in this review.
